# Measurement and Evaluation of Quantitative Performance of PET/CT Images before a Multicenter Clinical Trial

**DOI:** 10.1038/s41598-018-27143-4

**Published:** 2018-06-13

**Authors:** Yanjia Zhu, Caizheng Geng, Jia Huang, Juzhen Liu, Ning Wu, Jun Xin, Hao Xu, Lijuan Yu, Jianhua Geng

**Affiliations:** 1 Department of Molecular Imaging and Nuclear Medicine, Tianjin Medical University Cancer Institute and Hospital, National Clinical Research Center for Cancer, Key Laboratory of Cancer Prevention and Therapy, Tianjin Clinical Research Center for Cancer, Tianjin, 300060 China; 2 Department of Nuclear Medicine, Taizhou Hospital, #150 Ximen street, Linhai, Zhejiang province 317000 China; 3 Department of Nuclear Medicine, Zhejiang Jinhua Guangfu Hospital, Jinhua, 321000 China; 4 The People’s Hospital of Inner Mongolia Autonomous Region, Hohhot, 010017 China; 5 PET/CT Center, National Cancer Center/Cancer Hospital, Chinese Academy of Medical Sciences and Peking Union Medical College, Beijing, 100021 China; 6 Department of Radiology, Shengjing Hospital of China Medical University, Shenyang, 110004 China; 7 PET/CT-MRI Center, The First Affiliated Hospital, Jinan University, Guangzhou, 510630 China; 8 PET/CT-MRI Center, The Affiliated Tumor Hospital of Harbin Medical University, Harbin, 150081 China

## Abstract

To ensure the reliability of the planned multi-center clinical trial, we assessed the consistence and comparability of the quantitative parameters of the eight PET/CT units that will be used in this trial. PET/CT images were scanned using a PET NEMA image quality phantom (Biodex) on the eight units of Discovery PET/CT 690 from GE Healthcare. The scanning parameters were the same with the ones to be used in the planned trial. The ^18^F-NaF concentration in the background was 5.3 kBq/ml, while the ones in the spheres of diameter 37 mm, 22 mm, 17 mm and 10 mm were 8:1 as to that of the background and the ones in the spheres of diameter 28 mm and 13 mm were 0 kBq/ml. The consistency of hot sphere recovery coefficient (HRC), cold sphere recovery coefficient (CRC), hot sphere contrast (Q_H_) and cold sphere contrast (Q_c_) among these 8 PET/CTs was analyzed. The variation of the main quantitative parameters of the eight PET/CT systems was within 10%, which is acceptable for the clinical trial.

## Introduction

Multicenter study is a commonly used research method in clinical trial. Researchers can collect sufficient clinical data efficiently in this approach. However, to ensure the reliability of the research, the quality control (QC) process is usually crucial in these studies.

Quantitation of Standard Uptake Value (SUV) and related parameters play an important role in the diagnosis and treatment response evaluation using Positron Emission Tomography/Computed Tomography (PET/CT). However, it is well known that SUVs from different PET/CT units are of great discrepancy. The SUVs from different manufacturers are usually un-comparable. Fortunately, we (eight hospitals in China, installed Discovery PET/CT 690 from GE Healthcare within two years) agreed to initiate a multicenter clinical trial regarding the solid tumor treatment response evaluation using PET/CT.

Although we use the same PET/CT model, the discrepancy among different units tends to be larger than that in a single center, because of different experiment conditions, such as temperature, geography, and cross calibration. The assessment of the consistence and comparability of the quantitative data from these eight PET/CT units was the first step of our QC process. To the best of our knowledge, few researches on this have been done. Therefore we assessed the consistence and comparability of the quantitative data on these eight PET/CT units, based on NEMA NU2 2012^1^ standard, as the first step of the QC process of our multicenter clinical trial.

## Results

### Recovery coefficient

**Figure 1:**
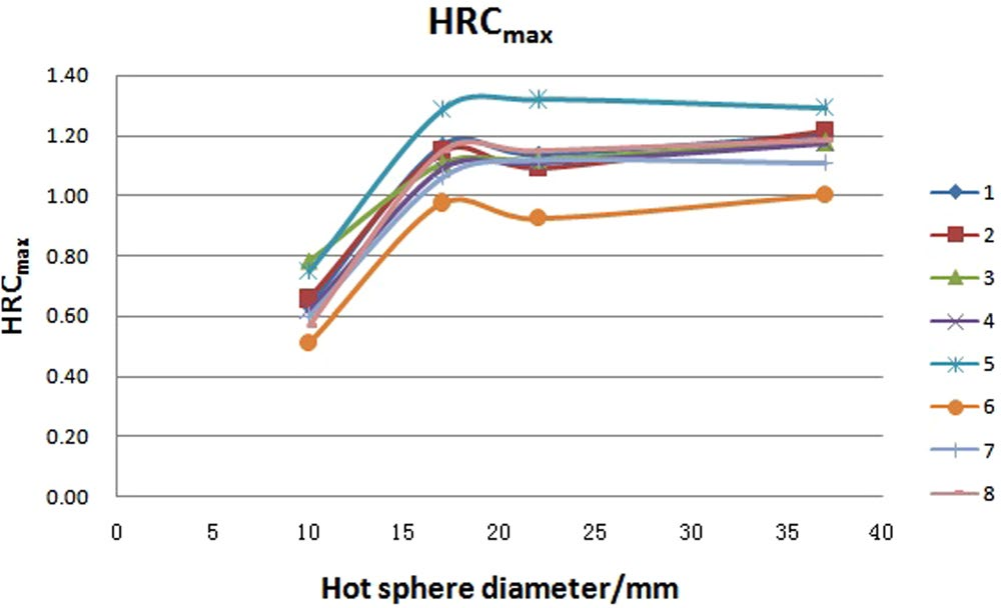
HRC_max_ distribution with spheres of different sizes in eight PET/CT systems. Notes: number 1 to 8 represents for the serial number of PET/CT systems.

**Figure 2:**
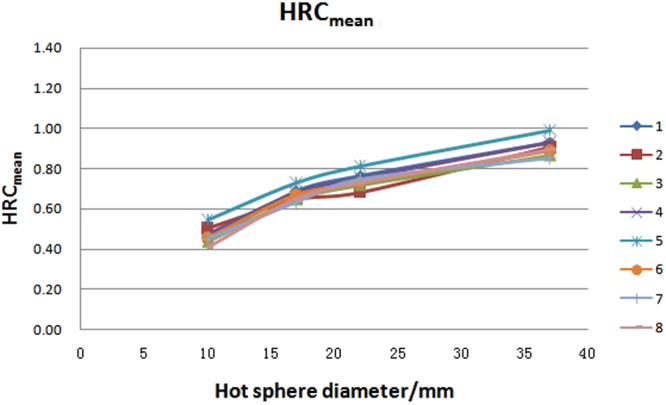
HRC_mean_ distribution with spheres of different sizes in eight PET/CT systems. Notes: number 1 to 8 represents for the serial number of PET/CT systems.

**Figure 3:**
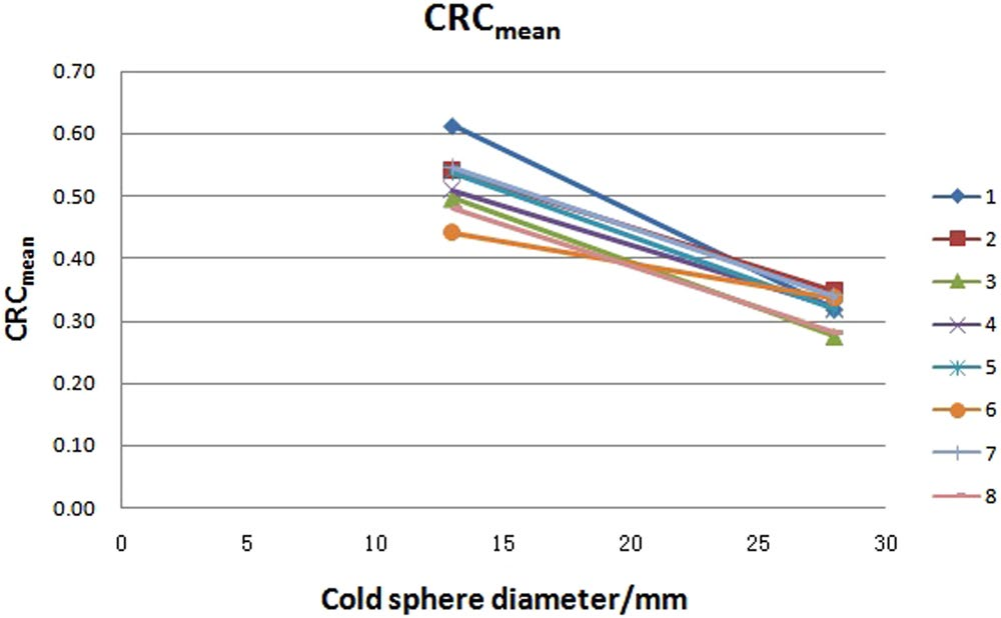
CRC_mean_ distribution with spheres of different sizes in eight PET/CT systems. Notes: number 1 to 8 represents for the serial number of PET/CT systems.

The HRC (hot sphere recovery coefficient) and CRC (cold sphere recovery coefficient), as well as the mean value, standard deviation (SD) value and variability (variability = SD/mean x 100%, generally named coefficient of variability, CV) of the eight PET/CT systems are presented in Table 1. The HRC and CRC distributions with different diameter of spheres are graphically shown in Figs 1–3. The variation of HRC_mean_ (HRC of mean uptake) and CRC_mean_ (CRC of mean uptake) were both within 10%. The corresponding value of HRC_max_ (HRC of maximum uptake) was less than 10% in hot lesions with diameter ≥17 mm, but more than 10% in hot lesions with diameter of 10 mm in the eight PET/CT systems. Along with the reduction of the sphere diameter, HRC_mean_ decreased, while CRC_mean_ increased. HRC_max_ increased along with the increasing diameter until 17 mm, and reached the plateau at 1 thereafter.

**Table 1:** HRC_mean_, HRC_max_, and CRC_mean_ in eight PET/CT systems.

PET/CT	37 mm	37 mm	22 mm	22 mm	17 mm	17 mm	10 mm	10 mm	28 mm	13 mm
number	HRC_mean_	HRC_max_	HRC_mean_	HRC_max_	HRC_mean_	HRC_max_	HRC_mean_	HRC_max_	CRC_mean_	CRC_mean_
1	0.93	1.2	0.77	1.14	0.69	1.17	0.44	0.64	0.32	0.61
2	0.9	1.22	0.68	1.09	0.65	1.15	0.5	0.66	0.35	0.54
3	0.87	1.18	0.72	1.12	0.65	1.11	0.44	0.78	0.27	0.5
4	0.93	1.17	0.75	1.11	0.68	1.09	0.48	0.62	0.32	0.51
5	0.99	1.29	0.81	1.32	0.73	1.29	0.54	0.75	0.32	0.54
6	0.89	1	0.73	0.92	0.67	0.97	0.46	0.51	0.34	0.44
7	0.85	1.11	0.75	1.12	0.63	1.06	0.45	0.6	0.34	0.55
8	0.89	1.19	0.73	1.15	0.65	1.15	0.41	0.57	0.28	0.48
Mean	0.91	1.17	0.74	1.12	0.67	1.12	0.46	0.64	0.32	0.52
SD	0.04	0.09	0.04	0.11	0.03	0.09	0.04	0.09	0.03	0.05
Variability/%	4.40	7.69	5.41	9.82	4.48	8.04	8.70	14.06	9.38	9.62

### Contrast

**Figure 4:**
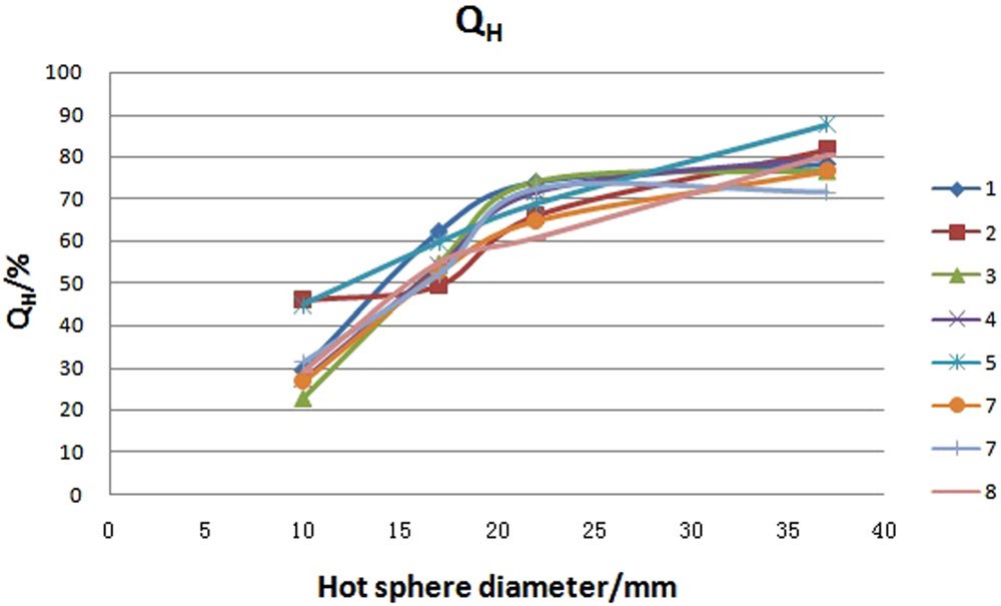
Q_H_ distribution with spheres of different sizes in eight PET/CT systems. Notes: number 1 to 8 represents for the serial number of PET/CT systems.

**Figure 5:**
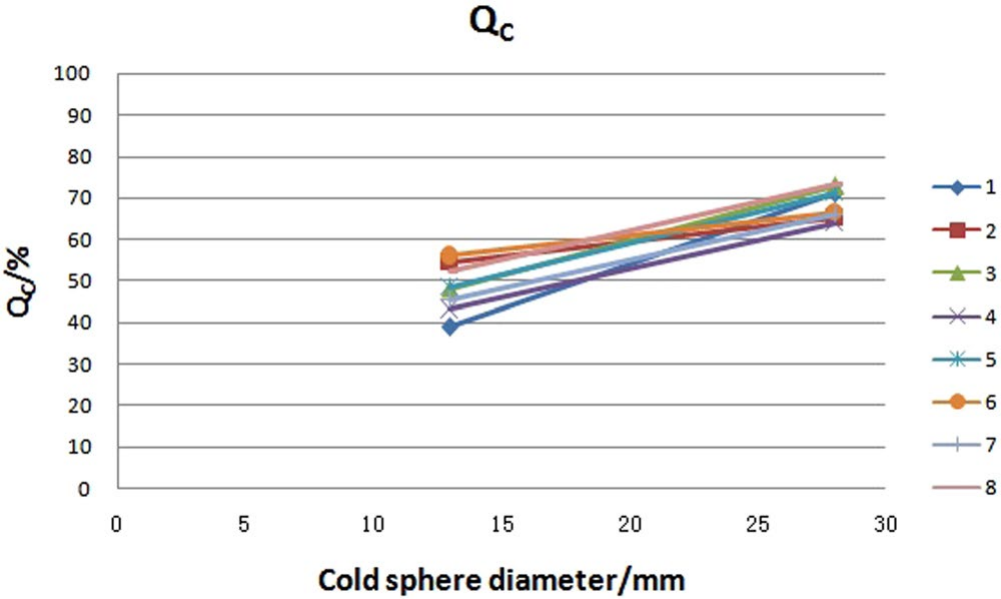
Q_c_ distribution with spheres of different sizes in eight PET/CT systems. Notes: number 1 to 8 represents for the serial number of PET/CT systems.

Q_H_ (hot sphere contrast) and Q_c_ (cold sphere contrast) were calculated by employing equations 4–5. The Q_H_, Q_c_, as well as the mean value, SD value and variability for the eight PET/CT systems are presented in Table 2. The Q_H_ and Q_c_ distributions with different diameter of spheres are graphically shown in Figs 4 and 5. The variation of Q_H_ was less than 10% in hot lesions with diameter ≥17 mm, but more than 10% in hot lesions with diameter of 10 mm. The variation of Q_c_ in 13 mm diameter sphere was also more than 10% in the eight PET/CT systems.

**Table 2:** Hot sphere contrast Q_H_ and cold sphere contrast Q_c_ in eight PET/CT systems.

PET/CT number	37 mm	22 mm	17 mm	10 mm	28 mm	13 mm
	Q_H_	Q_H_	Q_H_	Q_H_	Qc	Qc
1	78.12	73.87	62.23	29.76	70.91	38.77
2	81.69	66.06	49.45	45.95	65.22	54.57
3	76.74	74.08	54.76	22.83	72.73	48
4	79.94	71.71	53.98	27.31	63.81	43.09
5	87.63	68.87	59.8	44.98	71.07	48.35
6	76.51	64.79	52.65	26.89	66.37	55.97
7	71.69	72.49	52.25	31.38	66.09	45.39
8	80.28	60.78	55.03	29.38	73.28	52.08
Mean	79.08	69.08	55.02	32.31	68.69	48.28
SD	4.62	4.83	4.15	8.51	3.70	5.84
Variability/%	5.85	6.98	7.54	26.33	5.39	12.10

### Background variability

**Figure 6:**
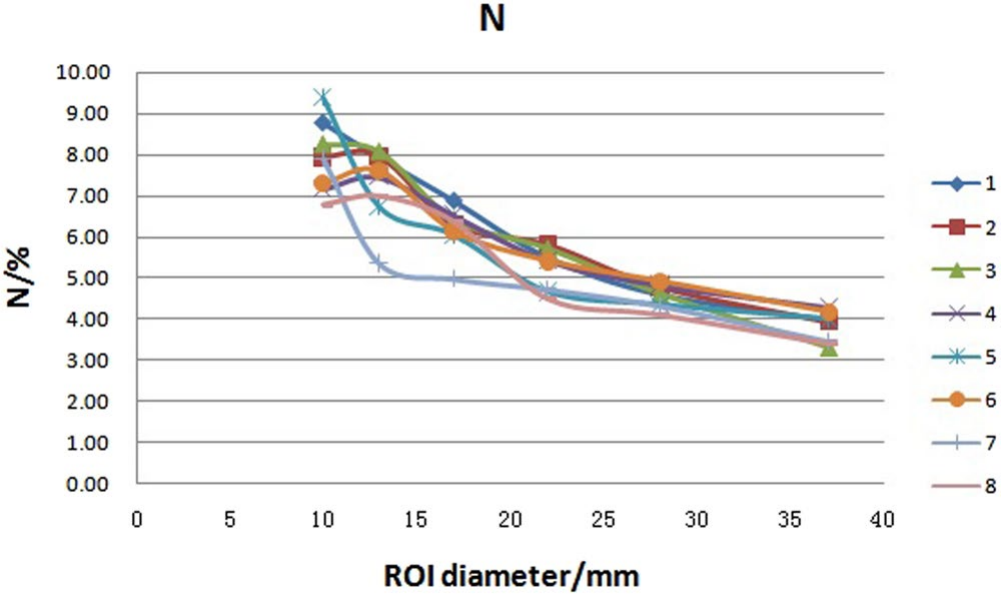
Background variability distribution with different ROI sizes in eight PET/CT systems. Notes: number 1 to 8 represents for the serial number of PET/CT systems.

Background variability (N) was calculated by employing equation 6. The result, as well as the mean value, SD value and variability in the eight PET/CT systems are presented in Table 3. The N distributions with different diameter of spheres are graphically shown in Fig. 6. The variation of N was less than 10% in hot lesions with diameter ≥17 mm, but more than 10% in hot lesions with diameter of 10 mm and in cold lesions with diameter of 13 mm.

**Table 3:** Background variability in eight PET/CT systems.

PET/CT number	37 mm	28 mm	22 mm	17 mm	13 mm	10 mm
	N	N	N	N	N	N
1	3.98	4.61	5.51	6.88	7.94	8.78
2	3.93	4.78	5.81	6.3	7.97	7.95
3	3.3	4.62	5.72	6.42	8.08	8.26
4	4.28	4.82	5.42	6.51	7.45	7.16
5	4	4.35	4.66	6.04	6.72	9.38
6	4.18	4.92	5.42	6.13	7.61	7.3
7	3.45	4.31	4.71	4.96	5.34	7.89
8	3.38	4.08	4.47	6.35	6.98	6.77
Mean	3.81	4.56	5.22	6.20	7.26	7.94
SD	0.38	0.29	0.52	0.56	0.91	0.87
variability/%	9.97	6.36	9.99	9.06	12.59	10.93

## Discussion

NEMA NU2 image quality phantom simulates the clinical hot and cold lesions and reflects the clinical image quality of PET/CT system. NEMA NU2 specifies two largest spheres (diameter of 37 mm and 28 mm) as the cold lesions and the other spheres (diameter of 22 mm, 17 mm, 13 mm and 10 mm) as the hot lesions. Considering the features of our planned clinical trial, to simulate the performances of hot tumor lesions with a variety of sizes, we used the two spheres with diameter of 28 mm and 13 mm for cold lesions and the others for hot lesions.

The recovery coefficient is a simple and intuitive indicator of partial volume effects. Due to the inherent limitations in theory and detection, the spatial resolution of PET is worse than CT (Computed Tomography) or MRI (Magnetic Resonance Imaging) system. This results in severe partial volume effect on PET images^2,3^. Ideally, recovery coefficient is 1 for hot lesions (most malignant tumors in PET) and 0 for cold lesions. However, the measured activity of hot lesion is usually lower than the actual value due to the partial volume effect. The cold lesion will lead to the opposite result. The smaller the size of the lesion is, the greater the partial volume effect will be^2,3^.

A study showed that when the sphere diameter is equal to three times the spatial resolution of the system expressed as the Full Width at Half Maximum (FWHM), the maximum pixel value of the image is theoretically 99.4% of the true value^2^. However, when the image is superimposed by noise, the measured activity can be higher than the actual one^3,4^. For a hot lesion whose size is close to the FWHM of the imaging system, the maximum activity concentration measured on the image can be reduced to 29% of the actual one^2^. This is consistent with our results. As the data showed in Table 1, in most cases, HRC_max_ results were larger than 1, except in the sphere with diameter of 10 mm. In addition, the variation of HRC_max_ was much larger than that of HRC_mean_. Therefore, the quantitative data related to the average value in the region-of-interest, such as SUV_mean_ or SUV_peak_^5^ will be favorable in the following clinical trial, instead of SUV_max_^6–9^. In our study, HRCmax increased along with the increasing diameter until 17 mm probably because of decreasing partial volume effect. When diameter became larger than 17 mm, HRCmax almost reached its theoretical value, i.e. 1. The fluctuation thereafter is mainly due to the noise in the image. A longer scanning time may help to mitigate the fluctuation.

Theoretically, there can be a platform area in the center of large lesions with no “activity loss”. The impacts of partial volume effect mainly on the edge of the lesion in this case and causes “activity loss”^2,3^. Our result showed that the HRC_mean_ of all hot spheres with diameter of 37 mm, 22 mm, 17 mm and 10 mm were smaller than 1. In addition, the smaller the hot lesion was, the lower the HRC_mean_ would be.

Partial volume effect, caused by the finite spatial resolution, is the main cause of error in PET quantification. As Figs 1 and 2 show, the lines of the eight units are almost parallel. This indicates that the variation among them was not from random error and statistical fluctuation of counts. This probably is caused by the differences in the accuracy of cross calibration between PET/CT system and the accessories used as well as the differences in spatial resolution in these units. Above all, the variation of the main quantitative data was within 10%, which is acceptable in the following multicenter clinical trial.

Our results showed also that the recovery capacity in small lesions was lower than the one in large lesions. In addition, the variation in small lesions among the eight units was larger. This will be an important cause of error in the following clinical trial. In this case, partial volume effect correction is necessary^10,11^.

With an ideal mean value of 100%, the contrast of the lesion in PET images reflects the relationship between the lesion and the surrounding background. This represents the overall image quality and the ability to detect small lesions of a PET system. As Table 2 and Figs 4 and 5 show, the contrast of hot and cold lesions decreased as the size of the lesion became smaller. The contrast of the biggest hot lesion (diameter 37 mm), and the smallest hot lesion (diameter 10 mm) were only 79.08% and 32.31% respectively. The smaller the lesion, the worse the detection ability of the device. This is consistent with other studies^12^. The contrasts were different among the eight devices. For hot spheres with diameter of 10 mm, the variation was up to 26.3%. This indicates that the small lesion detectability among these eight units are different. Therefore, more attention should be taken in the data analysis related to small lesion detectability in the following clinical trials.

Background variability reflects the noise level of the image. The greater the variability value, the higher the image noise. Our results showed that the background variability increased as the ROIs became smaller. The background variability is equal to the ratio of the SD to the mean value in the background ROI, and SD is equal to the square root of the mean value. In other words, the background variability is equal to the reciprocal of the square root of the mean value in the ROI^13^. When diameter of ROI was not more than 13 mm, the background variability among these eight devices was greater than 10%, suggesting that, in order to ensure the consistence in the following clinical trial, the background ROI should be as large as possible (diameter greater than or equal to 17 mm) when target-to-background ratio related parameters are investigated.

In summary, the variation of major quantitative performances was within 10% among the eight PET/CT units, which is acceptable for the following multicenter clinical trial. However, there are still some aspects that need to pay more attention in the setup of QC protocol before the clinical trial. Firstly, the quantitative data related to the average voxel value, such as SUV_mean_ or SUV_peak_ will be favorable, instead of SUV_max_. Secondly, for lesions with diameter ≤13 mm, the analysis of quantitative data needs to be careful. Thirdly, more attention should be taken in the data analyses related to small lesion detectability. Lastly, the background ROI should be drawn as large as possible when target-to-background ratio related parameters are investigated.

## Methods

### PET/CT facilities

Eight Discovery PET/CT 690 (GE Healthcare) scanners, installed within two years in China, were enrolled in this trial. Facilities are distributed in eight cities in China, from the northernmost one (Harbin, latitude 45.75 degrees) to the southernmost one (Guangzhou, latitude 23.16 degrees). Eight PET/CT scanners were numbered randomly from 1 to 8.

### Imaging

One NEMA image quality phantom (Biodex) of PET^1^ was used in this trial. The phantom internal length is 180 mm, and the cross section is in the shape of the human thorax. The longest inner diameters in the horizontal and vertical directions in the cross section are 294 mm and 224 mm, respectively. At the axial center of the phantom, a cylindrical insert with an outer diameter of 50 mm and an inner length of 180 mm was placed. Low density (0.3 g/ml) substance was filled into the cylinder insert to simulate the lung tissue. Six hollow spheres with thickness of 1 mm were evenly distributed around the lung insert. The inner diameters of these spheres were 37 mm, 28 mm, 22 mm, 17 mm, 13 mm and 10 mm, respectively.

To mitigate the operating discrepancy, one operator performed all the phantom preparation, imaging and data analysis procedures. The ^18^F-NaF concentration in the background was 5.3 kBq/ml, while the ones in the spheres of diameter 37 mm, 22 mm, 17 mm and 10 mm were 8:1 as to that of the background to simulate the hot lesion, and the ones in the spheres of diameter 28 mm and 13 mm were 0 kBq/ml to simulate the cold lesion.

The image acquisition and reconstruction protocols of the planned clinical trial will be the same with this investigation. PET images were acquired (2 beds, 2 min/bed), then reconstructed with Ordered Subset Expectation Maximization (OSEM) algorithm, 24 subsets, 2 iterations, and Gaussian post filter with full width at half maximum 6.4 mm, and with Time-of-Flight (TOF) and Point Spread Function (PSF) technologies. A z-axis 1:2:1 filter was used. The slice thickness of the reconstructed PET image was 3.27 mm. CT images were acquired with 140 kVp tube voltage and automatic tube current (15–180 mA), rotation speed 0.5 s and pitch of 0.984. Adaptive Statistical Iterative Reconstruction (ASiR, GE Healthcare) reconstruction algorithm was used for the reconstruction of CT data. The CT images were used for attenuation correction of PET images and image fusion with PET.

### Image analysis

The image analyses were performed using Xeleris Workstation (GE Healthcare). For recovery coefficient analysis, the sphere Volumes-of-Interest (VOIs) were delineated in the center of the sphere on PET images. The radioactive uptake concentration (kBq/ml), maximum pixel counts in hot lesions (C_max,H,j_), average pixel counts in hot lesions (C_mean,H,j_), as well as in cold lesions (C_mean,C,j_) and their SDwere assessed, where j represents for the number of the sphere.

For contrast and background variation analyses, we used the method of NEMA NU2^1^ standard. The Regions-of-Interest (ROIs) were delineated on the transaxial CT images in the center of the spheres, and then projected on the PET images. The mean counts (C_B,j_) and SD_B, j_of ROIs with the same diameter were recorded.

HRC and CRC were calculated by employing Equations 1–3 ^14,15^. While Q_H_, Q_c_and background variability (N) were assessed by employing Equations 4–6 ^1^.

1 $${{\rm{HRC}}}_{{\rm{\max }},{\rm{j}}}={{\rm{C}}}_{{\rm{\max }},{\rm{H}},{\rm{j}}}/{a}_{H}\ldots $$

2 $${{\rm{HRC}}}_{{\rm{mean}},{\rm{j}}}={{\rm{C}}}_{\mathrm{mean},H,j}/{a}_{H}\ldots $$

3 $${{\rm{CRC}}}_{\mathrm{mean},j}={{\rm{C}}}_{{\rm{mean}},{\rm{C}},{\rm{j}}}/{a}_{B}\ldots $$

4 $${{\rm{Q}}}_{{\rm{H}},{\rm{j}}}=({{\rm{C}}}_{{\rm{mean}},{\rm{H}},{\rm{j}}}/{{\rm{C}}}_{{\rm{B}},{\rm{j}}}-{\rm{1}})/({{\rm{a}}}_{{\rm{H}}}/{{\rm{a}}}_{{\rm{B}}}-{\rm{1}})\times 100 \% \ldots $$

5 $${{\rm{Q}}}_{{\rm{C}},{\rm{j}}}=({\rm{1}}-{{\rm{C}}}_{{\rm{mean}},{\rm{C}},{\rm{j}}}/{{\rm{C}}}_{{\rm{B}},{\rm{j}}})\times {\rm{100}} \% {\rm{\ldots }}$$

6 $${\rm{Nj}}=({{\rm{SD}}}_{{\rm{B}},{\rm{j}}}/{{\rm{C}}}_{{\rm{B}},{\rm{j}}})\times {\rm{100}} \% \ldots $$

### Ethics Statement

Only phantom study is included in this study, so basically the ethics statement is not necessary.
